# Contribution of Clinical Nurses to Hospital Efficiency and Economic Sustainability: A Systematic Review

**DOI:** 10.1155/jonm/3332688

**Published:** 2025-03-19

**Authors:** Daniel Bárcenas-Villegas, Rocío Cáceres-Matos, Soledad Vázquez-Santiago

**Affiliations:** Nursing Department, Faculty of Nursing, Physiotherapy and Podiatry, University of Seville, Avenzoar, 6 Street, Seville 41009, Spain

**Keywords:** cost-benefit analysis, healthcare expenditure, hospital administration, hospital economics, nurse administrator, registered nurses

## Abstract

**Aim:** To analyze the existing evidence on the contribution of the nursing profession to efficiency and healthcare sustainability in the hospital setting.

**Background:** Promoting economic efficiency in hospital centers is a key factor in ensuring their long-term sustainability. In this regard, nursing professionals with caregiving roles could emerge as valuable contributors to the survival of the existing hospital model.

**Design:** A systematic review was conducted following PRISMA guidelines. This review has been registered with PROSPERO under the registration number: CRD42023481140.

**Method:** A search was conducted across four international databases (CINAHL, PubMed, Scopus, and WOS) from 2013 to the present for studies in English and Spanish. Primary studies on economic evaluations and systematic reviews were included. The study selection was carried out in three stages, with two reviewers independently analyzing the data and resolving disagreements through discussion and consensus. The quality assessment utilized the CASP tool, the CHEERS checklist, and the STROBE statement.

**Results:** Out of 3058 records identified, nine were deemed eligible, comprising one longitudinal study, four descriptive studies, two systematic reviews, two randomized controlled trials, one cohort study, and two case-control studies, with a total of 333,597 patients. The studies varied in sample size, intervention strategy, content, measurement scales, and statistical analysis of the primary outcome. The studies indicate that health education provided by hospitals is cost-effective, potentially generating costs below 100,000 dollars per quality-adjusted life year. Investing in nursing specialty, advanced practice nurses, and clinical safety reduces the number of admissions and decompensations.

**Conclusions:** This review highlights that health education and clinical safety are the areas where nursing tasks have the most significant economic impact. Nursing specialty and the inclusion of advanced practice nurses are proving to be fields towards which health systems should focus to promote a more economically sustainable model.

## 1. Introduction

Nowadays, it is complex to discuss healthcare without considering the economic aspect; this duality is inseparable in any healthcare organization, encompassing everything from human to material resources [1]. In this context, some authors coin the term “economic evaluation” to conduct a comparative analysis of costs and effects aimed at health as a determinant for analyzing the sustainability and efficiency of an organization [1, 2].

To conduct economic evaluations, cost-minimization analysis is used, comparing the costs of two interventions assuming mutual benefit, along with cost-effectiveness analysis, where the benefits of strategies are measured in units of morbidity, mortality, or quality of life; cost-utility analysis, where the quality and quantity of life are considered beneficial; and cost-benefit analysis, where monetary terms are used to express the consequences of interventions [3].

Providing quality healthcare is associated with resource consumption that must respond to an ever-changing reality. In this sense, “the greater complexity faced by healthcare organizations reflects new needs and expectations of citizens, leading to the incorporation of numerous variables in management, such as social factors, transparency, and sustainable development” [4]. Nurses become one of the healthcare professionals with the most significant impact on the health economy due to the increase in chronic diseases, the scarcity of hospital resources, and the trend towards a model that aims to promote independence and humanization [1]; Rogowski, 2024.

Furthermore, the increasing demand for care today is difficult to address with the current healthcare model. Aspects such as home hospitalization and the creation of Magnet hospitals are proving to be an alternative to the traditional biomedical model, presenting a more comprehensive, human, and integrated approach to patients in their health-illness process [5–7].

Baby, John, and Thomas [8] and Soyemi and Aborode [9] state that the home care model has gained strength in recent years due to hospital bed overoccupancy, added to the increase in chronic and degenerative diseases.

In this scenario, the nursing profession must be positioned as an alternative to the classical healthcare culture [9], leading an efficient management model and empowering the healthcare staff, whose tradition is marked by high management and leadership capacities in an informal manner [10, 11].

In this sense, the clinical nurse is profiled as a professional with excellent curricular training and skills, becoming a key element for achieving and maintaining healthcare efficiency [6, 7]. Given the existing scientific evidence, we pose the following research question: Are the activities of clinical nurses efficient and sustainable for hospitals? Therefore, the objective of this work is to analyze the existing evidence on the contribution of the nursing collective to healthcare efficiency and sustainability.

## 2. Method

### 2.1. Design and Search Strategy

A systematic review of the literature was conducted following the recommendations of the Preferred Reporting Items for Systematic Reviews and Meta-Analyses (PRISMA) Checklist statement [12, 13]. Five international databases were reviewed (Cumulative Index to Nursing and Allied Health Literature [CINAHL], Pubmed, SCOPUS, Web of Science [WOS], and Cochrane Database of Systematic Reviews) by two independent researchers between October and December 2023. In case of disagreement between reviewers, it was resolved by consensus with a third researcher. The systematic review protocol was published in the International Prospective Register of Systematic Reviews (PROSPERO) with the registration number: CRD42023481140.

The search strategy used Medical Subject Headings (MeSH) and Boolean operators, as well as their Spanish equivalents, adapted to each database as follows: (“Cost Effectiveness” OR “Economic evaluation” OR “Economics, hospital) AND (“nurse⁣^∗^ OR “Nursing”) AND “hospital⁣^∗^.”

### 2.2. Selection of Studies and Eligibility Criteria

Studies that met the following inclusion criteria were considered for the review: (1) articles relating nursing work with elements of effectiveness or efficiency and cost (efficiency) in the public and private hospital setting; (2) studies on partial or complete economic evaluations; (3) with the methodology of clinical trials, quasiexperimental studies, observational studies, and descriptive studies; (3) in Spanish and/or English language; and (4) published from 2012 to the present.

Exclusion criteria comprised the following criteria: (1) studies not focusing on nurse-led or nurse-promoted interventions, (2) conducted in primary care, (3) not addressing the contribution of nurses in terms of costs, and (4) studies with methodological deficiencies.

### 2.3. Screening and Data Extraction

Two reviewers conducted screening and data extraction independently, resolving any disagreements through discussion and consensus, involving three stages. In the initial phase, reviewers evaluated the titles and abstracts according to the search strategy, aiming to identify studies that potentially met the established selection criteria. In the second stage, the relevance of the studies was determined based on the inclusion and exclusion criteria defined in the protocol, using the full texts of the studies.

Records with methodological deficiencies (such as unidentified study types, objectives, results, and conclusions) and quality issues (lack of quality assessment or failure to meet minimum evaluation criteria) were subsequently excluded. This process resulted in the final selection of articles for review.

For data extraction, a double-entry table was designed, including sections for the instrument name, version and year, sample size, content, criterion validity, reliability, and construct validity. Two reviewers independently collected data, discussing their findings with a third reviewer in case of discrepancies.

### 2.4. Quality Assessment of the Included Studies and Risk of Bias (ROB)

For assessing the quality of the studies, the CASP tool [14] was used. This tool follows a structure that evaluates validity, results, and applicability, whereas the score increases and the study's quality improves. The Strengthening the Reporting of Observational Studies in Epidemiology (STROBE) statement [15] was used for evaluating observational studies. This tool is employed to ensure clear and comprehensive reporting of observational studies, and in this review, it was applied to studies where the CASP tool could not be used.

In addition, the Consolidated Health Economic Evaluation Reporting (CHEERS) checklist [16] of 28 items was employed to report the quality of health economic evaluations, where none indicates the absence of measurement, N/A denotes unclear measurement, and one indicates present measurement.

For assessing the ROB, the ROB 2 tool (Table 1) was utilized. This tool measured the process of randomization (D1), deviations from intended interventions (D2), missing outcome data (D3), outcome measurement (D4), and selection of reported results (D5). The level of bias risk was measured using symbols such as “+” (*high risk*), “−” (*low risk*), and “!” (*concerns*).

## 3. Results

### 3.1. Selection of Studies Included in the Systematic Review

**Figure 1:**
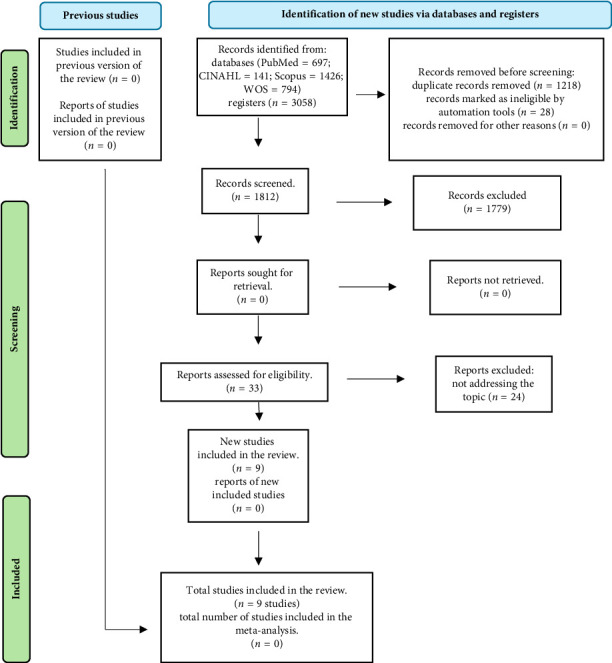
Flowchart of the identification and screening process of the included studies. Note. Fountain: the PRISMA 2020 statement: an updated guideline for reporting systematic reviews of [12] BM.

The database search yielded an initial identification of 3058 articles, which was reduced to a total of 1812 after removing duplicates. After evaluating titles and abstracts, 33 full-text documents were reviewed, of which nine studies were ultimately included (16 studies were excluded for not clearly meeting the established inclusion criteria, eight for methodological deficiencies, and four for quality deficiencies). The flow diagram illustrates in detail the search and selection process of the included studies (Figure 1).

### 3.2. Characteristics of the Included Studies

Table 1 provides a detailed description of the main characteristics of each of the incorporated studies, including authors and publication year; study type, population, and country of origin; main objective, grouping category, and analysis conducted; as well as the theme and main results. In this regard, the studies were grouped into four themes: (1) health education, (2) specialist and advanced practice nurses, (3) clinical safety, and (4) clinical process management.

### 3.3. Quality Assessment of the Included Studies

Overall, the articles analyzed reported good quality according to the tools employed. Observational studies demonstrated high quality in their analysis, although Moreton et al. [20] and Wieczorek-Wójcik et al. [25] did not meet as many of the measured items, failing to specify confidence intervals, exposure–outcome relationship, and sensitivity. Conversely, the studies by Ward et al. [24] and Driscoll et al. [17] achieved the highest scores in the CASP analysis. The quality of the studies is presented in Table 2.

In the assessment of the quality of economic evaluations (CHEERS), good quality was generally evident. Of all the articles analyzed, articles by Murphy et al. [21] obtained the lowest scores; however, although the remaining articles did not achieve the maximum score in the measurement tools used, they all maintained a high level of compliance with the items assessed. The studies by Moreton et al. [20] and Noble et al. [22] stood out as demonstrating a higher quality than others in the evaluation process.

### 3.4. Assessment of ROB in the Included Studies

A high ROB was identified among the analyzed studies in Table 3, with works by Wieczorek-Wójcik et al. [25] standing out as those with lower ROB. The randomization process was scarce, with five articles notable for this [17, 19, 21, 22]. Other evaluated aspects, D2, D3, and D4, showed variations in results regarding bias risk, with D5 being notable for articles raising major concerns about this risk [17–19, 21, 22].

### 3.5. Main Findings From the Included Studies

The included studies primarily analyzed four areas in which the nurse's role impacts cost-effectiveness. Two studies focussed on health education [18, 24], four on the role of specialist and advanced practice nurses (APNs) [17, 20, 22, 23], four on clinical safety [17, 19, 21, 25], and one on clinical process management [23].

Regarding the studies focusing on the economic impact of health education, Ward et al. [24] highlighted the importance of health education in addressing smoking cessation, a high economic impact area for hospitals through a randomized trial. The average cost per patient in the nurse-led intervention group was $173.61, which was significantly higher than in the usual care control group ($67.80). Medication costs were also higher in the intervention group ($78.78 in the intervention group vs. $62.32 in the control group). However, the nurse-led intervention group demonstrated a smoking cessation rate of 15.7%, compared to 7.0% in the control group. In addition, the incremental cost-effectiveness ratio (ICER) was $1325 per quit, indicating that the cost per patient who quit smoking was $1325, with a 99.9% probability of being cost-effective when considering a threshold of $5000 per quit.

Lai et al. [18] also identified the implementation of health programmes by nurses as a crucial aspect for efficient hospital cost management, noting significant reductions in service expenses and hospital readmission rates. This study evaluated the cost-effectiveness of nurse-led visits compared to the routine care group. It was found that patients in the nurse-led group made fewer visits to the emergency department compared to the traditional intervention (2.18 times fewer visits). However, the cost for the intervention group was slightly higher than that of the traditional group (intervention group £504 vs. control group £860). Despite this, the results indicated that the programme was cost-effective in terms of incremental cost per quality-adjusted life year (QALY).

Regarding the role of specialist and advanced practice nurses, authors such as Twigg et al. [23], Noble et al. [22], Moreton et al. [20], and Driscoll et al. [17] agree on the positive impact of interventions by specialist and APNs on healthcare economics. Twigg et al. [23] assessed the cost-effectiveness of increasing nursing care hours in adults in Western Australia. The results showed that the implementation of the “nurse hours per patient day” (NHPPD) method was cost-effective, with an incremental cost of AUD$8907 per life year gained. In this regard, the accepted cost-effectiveness threshold in Australia ranged from AUD$30,000 to AUD$60,000 per quality-adjusted life year gained. Although the intervention incurred an additional cost of AUD$16,833,392 due to the increase in nursing hours, the savings from the reduction in adverse events were substantial. This demonstrates that investing in nursing staff not only improves clinical outcomes but is also a cost-effective strategy for healthcare systems.

Noble et al. [22] found that specialist nurses improve economic efficiency by successfully intervening in clinical settings. In this study, no significant effect of the nurses' efforts was found in reducing emergency department visits over the past 12 months, compared to the usual care group. On the contrary, hospital stays were shorter in the intervention group, which reduced overall hospitalization costs (£2202 in the intervention group vs. £2948 in the control group). In terms of QALY, the ICER was £26,445 per additional QALY if the nurse-led intervention was not used, indicating that the intervention was not cost-effective in QALY terms.

Similarly, Moreton et al. [20] emphasized the reduction of readmissions and exacerbations in frail and palliative patients as crucial factors in resource management when specialist nurses lead care. Patients in the nurse-led group were less likely to die in the hospital, and fewer hospital readmissions were reported compared to the traditional group. In addition, the estimated cost of hospital stays was reduced by half. Driscoll et al. [17] evaluated the cost-effectiveness of a nurse practitioner–led specialized service, finding that it was both more economical and effective compared to the usual care system (intervention group $23,031 vs. $25,111 in the usual care group).

Clinical safety emerges as a significant factor for hospital economics; indeed, for Murphy et al. [21], Lasater et al. [19], and Wieczorek-Wójcik et al. [25], the need to adjust nurse-to-patient ratios is essential to improve hospital efficiency. According to Murphy et al. [21], this adjustment of nurse-to-patient ratios is associated with a reduction in costs from adverse events, resulting in significant savings both annually and in acute care settings. Lasater et al. [19] linked these ratios to a decrease in hospital readmissions and a reduction in hospital stays. Lastly, Wieczorek-Wójcik et al. [25] noted that increased spending on nurses with higher qualifications is associated with a reduction in mortality rates in nonsurgical units.

Finally, clinical process management from the nursing perspective emerges as a crucial element for sustainability and healthcare efficiency. According to Twigg et al. [23], implementing the “NHPPD” method is economically viable compared to traditional management methods, leading to a significant improvement in quality of life. The intervention led to the prevention of 1357 nursing-sensitive adverse events, such as surgical wound infections, pulmonary failure, and cardiac arrests, and resulted in a gain of 1088 QALYs.

## 4. Discussion

The findings of this study revealed that nurses' contribution to the efficiency and sustainability of hospitals extends beyond purely clinical realms. Specifically, it was observed how nurses have a positive impact on hospital economics through the implementation of health education programmes, integration of specialist nurses and APNs, optimization of clinical processes, and enhancement of clinical safety by adjusting nurse–patient ratios. In fact, all authors in the reviewed articles underscore the importance of bedside nurses in healthcare economics; this is noteworthy because, despite being studies from countries with similar quality-of-life situations, they represent different healthcare models that yield similar results regarding economic efficiency.

Regarding the resulting themes, one of the most relevant aspects with a significant impact on health economics relates to health education. Although this is currently considered by most healthcare services with the inclusion of self-care promotion programmes, we believe that there should be an even greater emphasis on promoting this area of work among nurses to opt for a healthcare model with better cost outcomes and thus a greater future viability.

Teaching issues such as self-care and self-management of clinical processes help reduce hospital pressure and the number of readmissions at the time when the availability of hospital beds is decreasing [26, 27].

On the other hand, one of the aspects that the nursing profession is increasingly focusing on is the inclusion of APNs in the human resource structure. While historically nurses have been seen as generalists except for some specialty such as midwifery, the administration itself has hindered the promotion of more nursing specialty. In this regard, APNs emerge as the possibility of having nurse leaders in specific processes (chronic wounds and heart failure, among others), which seems to open an alternative path to nursing capacities.

For authors such as Egerod et al. [28], Horvath et al. [29], and Jiménez-García et al. [30], this has been shown to reduce relapses and hospital readmissions, resulting in improved healthcare costs. In this sense, Salamanca-Balen et al. [31] detailed how nurse-led interventions promote the efficient use of specific resources, such as hospitalizations, length of stay, and care costs, contributing to overall hospital efficiency. On the contrary, examples of this are the findings of Bryant-Lukosius et al. [32], where transition treatments by specialist nurses did not generate economic benefits or improvements in health outcomes for oncology and cardiac surgery patients.

Another area with a direct influence on hospital economics stems from nurse-to-patient ratios. According to authors such as Ayuso-Fernandez et al. [33] and Fernández-García [34], adjusting nurse–patient ratios not only enhances care quality but also affects morbidity and mortality rates. Although this issue has been raised for years, there is still little awareness from administrations to regulate this issue.

Finally, one aspect closely related to our country's current healthcare situation is the possibility for nurses to manage clinical processes. Currently, in Andalusia and primary care settings, efforts are being made in this regard with the resolution of processes from the nursing reception consultation. This offers the opportunity for nurses to manage patient referrals to hospitals, streamline delays in health centers, and even resolve noncomplex clinical processes. The latter does not constitute an intrusion into the field of medicine but rather situations that arise, for example, in emergencies and do not require medical personnel for resolution, such as unblocking a urinary or nasogastric catheter, simple wound care, or even simple processes where nurses can act under exhaustive protocols. In addition, when this issue has been addressed out of necessity, as was the case during the COVID-19 pandemic, nurses' process management tasks represented a turning point without which the same success would not have been possible [27, 35].

Regarding the quality of the studies, according to the evaluation performed using the CASP method, CHEERS checklist, and STROBE statement, the selected articles exhibit an overall high level of quality, although there is a notable diversity among them.

## 5. Limitations and Strengths

Several limitations of this study must be considered when generalizing the findings. First, the diverse geographical locations and varying approaches used to evaluate nurses' contribution to hospital efficiency across different studies may hinder the extrapolation of results to our hospital setting. Second, some articles highlight the need for larger sample sizes, potentially impacting the results [18, 20, 22]. Third, there appears to be a shortage of studies directly linking clinical nurses to economic efficiency, with a prevalence of research focusing on technical and direct care aspects.

Regarding the strengths of this research, it is important to note that most reviewed articles exhibit a high level of quality according to various analytical methods employed. This consistency in study quality provides a solid foundation for the conclusions drawn in this analysis. Furthermore, despite the diversity in the typology of included research in this study, it is noteworthy how the results are largely similar. This coherence in findings suggests consistency in nurses' contribution to healthcare efficiency, regardless of differences in contexts or methodological approaches.

Moreover, the geographic diversity in which the analyzed studies were conducted constitutes another significant strength. This variety of geographical locations allows for a global and varied view of how nurses contribute to healthcare efficiency in different environments and health systems. By considering studies conducted in different countries or regions, common patterns can be identified, and conclusions can be adapted for practical applications in diverse contexts.

Furthermore, analyzing existing literature on nurses' contribution to hospital efficiency can offer valuable insights and knowledge for decision-making in health policies and human resource management in the healthcare sector. By better understanding nurses' roles and interventions in different contexts, policymakers can develop more effective strategies to improve healthcare efficiency and quality.

In conclusion, there is a need for greater investment in health education programmes led by nurses; the integration of specialist nurses and APNs can significantly contribute to reducing readmissions and improving costs; it is essential for administrations to recognize the importance of regulating nurse-patient ratios to enhance care quality and health outcomes; finally, nurses' ability to manage clinical processes can be an effective strategy to streamline care and improve outcomes, especially in emergency situations such as the COVID-19 pandemic.

## 6. Conclusions

Nurses with clinical responsibilities form a professional group that positively impacts hospital's economic efficiency. Health education and clinical safety are the areas where nursing tasks are most influential on the economy, as they directly reduce readmissions, relapses, and even deaths. Nursing specializations and the inclusion of APNs are proving to be fields that healthcare systems should focus on to promote a more economically sustainable model. Given that nurse-led clinical process management in primary care has reduced waiting lists and efficiently resolved consultations, it is essential to highlight this working methodology in the hospital setting as a management measure aimed at quality and efficiency. Finally, more studies are needed to address the role of nurses and their direct impact on healthcare economics.

## Figures and Tables

**Table 1:** Characteristics of the articles included in the review.

Author/year	Type of study Population Country Sample size	Objective Thematic Analysis	Topic	Main results
Driscoll et al. [17]	Economic evaluation. 500 Patients admitted for exacerbation of heart failure from a Melbourne hospital. Australia	To determine the cost-effectiveness of a hospital heart failure service with specialist nurses compared to usual practice. Nurse practitioners and advanced practice nurses' cost-effectiveness analysis quality of life value	Impact on costs and quality of life of nurse specialists in patients with heart failure	After 12 months of study, the group that had specialist nurses saved more than $2000. QALY was better in the group of patients with specialist nurses. Incremental cost-effectiveness resulted in savings of $109,474 per QALY at 12 months
Lai et al. [18]	Randomized controlled trial. 124 patients. China	To assess the cost-effectiveness of a nurse-led care programme for breast cancer patients who received outpatient chemotherapy. Health education cost-utility analysis	Importance of the development of nurse-led programmes for the health of cancer patients and for healthcare costs	For people who received series cycle chemotherapy, the estimated number of emergency room visits was 2188 times higher for the routine care group compared to the directed care group. The incremental cost-utility ratios were 8856 pounds and 18,936 pounds per quality-adjusted life year gained for those who received four- and six-cycle chemotherapy
Lasater et al. [19]	Observational. 87 intensive care hospitals and a total of 210,493 patients. USA	To analyze changes in nursing staffing ratios in Illinois hospitals. The aim of this study is to determine whether a higher workload for nurses is linked to mortality, length of stay, and hospital costs. Clinical safety cost-benefit analysis	Impact on patients and costs of nurse–patient ratios in hospitals	After adjusting for nurses per patient, the probability of 30-day mortality for each patient increased by 16% for each additional patient workload. If the hospitals in the study had a staffing ratio of 4:1 during the 1 year study period, more than 1595 deaths would have been prevented and more than $117 million would have been saved
Moreton et al. [20]	Observational/economic evaluation. Palliative care unit patients and caregivers (321 patients) Australia	To assess the clinical, economic, and personal impact of nurse specialists in a community-based palliative care service at Sydney Adventist Hospital. Nurse practitioners and advanced practice nurses' cost-effectiveness analysis	Importance of specialized nursing work in the hospital environment and its impact on the management of palliative patients in the community environment	With the new implementation, the service halved the estimated cost of hospitalization per patient. The length of hospital stay was not affected. Estimated cost per patient was reduced by 51.9%. The number of hospital admissions was lower since the intervention
Murphy et al. [21]	Cohort 5544 patients discharged. Ireland	Identify the costs associated with adverse events sensitive to clinical safety retrospective analysis	Impact on adverse events experienced by nurses at current rates	16% of the sample had an adverse event associated with nurses. The economic cost of nurse-sensitive adverse events was estimated at €91.3 million per year. This study paves the way for the study of nurse ratios with the aim of improving clinical safety
Noble et al. [22]	Clinical trial/economic evaluation. 85 adults with epilepsy. United Kingdom	To analyze the cost-effectiveness of employing epilepsy nurses in patients with this condition. Nurse practitioners and advanced practice nurses' cost-effectiveness analysis	Influence of nurses specializing in chronic clinical processes that generate hospital admissions and influence the costs of the system	The inclusion of epilepsy nurses saved £558. The costs associated with conventional intervention without a specialist were 527% higher. Although the costs of specialist nurse interventions were lower, so was the QALY of patients
Twigg et al. [23]	Longitudinal/economic evaluation of patients from 3 hospitals (107,026 patients). Australia	To assess the economic impact of increased nursing care hours on health outcomes in adult teaching hospitals. Clinical process management retrospective/cost-effectiveness analysis	Benefits for patients of the incorporation of more nurses in care and its relationship with healthcare costs	Nursing-sensitive outcomes were 1357 fewer than expected after implementation and included 155 fewer rescue failure events. The total number of years of life gained by quality was 1088. The cost per year of life gained was $8907
Ward et al. [24]	Randomized trial/economic evaluation. Smoking patients from 5 Michigan hospitals (1370 patients). USA	To assess the cost-effectiveness of inpatient smoking cessation interventions. Health education cost-effectiveness analysis	Nurse health education for hospitalized tobacco users	The cost per person for the new smoking cessation services was higher than for the usual intervention. The incremental propensity-adjusted cost ratio was $1325 for quitting smoking with a 99.9% cost-effectiveness
Wieczorek-Wójcik et al. [25]	Cases and controls/economic evaluation. Adults hospitalized in 4 nonsurgical wards (6 + 5031 patients). Poland	To assess the cost-effectiveness of employing nurses with higher education. Nurse practitioners and advanced practice nurses' cost-effectiveness analysis	Effect on the economy and health of increased dedication to nurse education	The cost of increasing the percentage of nurses with the most education by 1 h amounted to $1.95. The incremental change resulted in a reduction of 1.01/1000 patients. The cost-benefit analysis resulted in a gross profit of $14,790,547.93

*Note:* Own development.

**Table 2:** Analysis of articles' quality.

Article	Quality		Reporting checklist	Description of the analysis
	CASP	CHEERS	STROBE	
Twigg et al. [23]	Economic evaluation (7/12)	(19/28)	(21/22)	Confidence intervals for results are not specified
Noble et al. [22]	Economic evaluation (6/12)	(24/28)		There is no random sample selection
Lai et al. [18]	Randomized controlled trial (9/11)	(24/28)		No blinding of participants or assessors was carried out
Moreton et al. [20]	Economic evaluation (9/12)	(24/28)	(15/22)	The sensitivity of the analysis is not carried out
Ward et al. [24]	Economic evaluation (11/12)	(23/28)		There is no random sample selection.
Lasater et al. [19]	Cross-sectional studies (8/11)		(22/22)	All the sections to be evaluated are adequately detailed
Murphy et al. [21]	Economic evaluation (7/12)	(18/28)	(22/22)	Confounding factors have not been taken into account
Wieczorek-Wójcik et al. [25]	Economic evaluation (9/12)	(21/28)	(15/22)	We do not know the relationship between exposure and outcome. Confidence intervals are not reflected. The results cannot be applied to our environment
Driscoll et al. [17]	Economic evaluation (11/12)	(23/28)		The results cannot be applied to our environment

*Note:* Own development.

**Table 3:** Risk of bias.

Articles	D1	D2	D3	D4	D5	General
Twigg et al. [23]	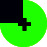	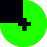	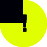	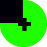	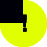	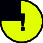
Noble et al. [22]	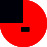	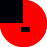	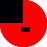	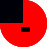	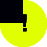	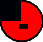
Lai et al. [18],	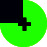	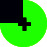	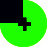	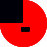	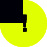	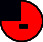
Moreton et al. [20]	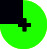	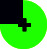	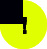	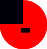	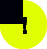	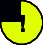
Ward et al. [24]	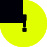	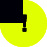	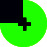	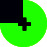	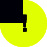	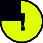
Lasater et al. [19]	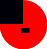	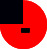	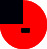	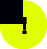	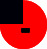	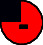
Murphy et al. [21]	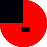	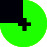	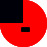	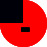	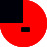	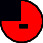
Wieczorek-Wójcik et al. [25]	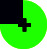	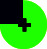	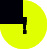	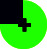	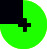	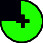
Driscoll et al. [17]	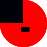	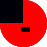	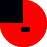	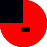	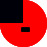	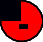

*Note:* Own development. D1, random measurement; D2, deviations from planned interventions; D3, missing result; D4, outcome measurement; D5, selection of the reported outcome.

## Data Availability

The data from the present study are available for production.
