# Correction to “Contribution of Clinical Nurses to Hospital Efficiency and Economic Sustainability: A Systematic Review”

**DOI:** 10.1155/jonm/9765945

**Published:** 2025-09-25

D. Bárcenas-Villegas, R. Cáceres-Matos, and S. Vázquez-Santiago, “Contribution of Clinical Nurses to Hospital Efficiency and Economic Sustainability: A Systematic Review,” *Journal of Nursing Management* 2025 (2025): 3332688, https://doi.org/10.1155/jonm/3332688.

In the article titled “Contribution of Clinical Nurses to Hospital Efficiency and Economic Sustainability: A Systematic Review,” author order in the author list was incorrect, where:

**Daniel Bárcenas-Villegas, Rocío Cáceres-Matos, Soledad Vázquez-Santiago**

Should have read:

**Daniel Bárcenas-Villegas, Soledad Vázquez-Santiago, Rocío Cáceres-Matos**

We apologize for this error.

